# Spatio-Temporal Evolution of Sporulation in *Bacillus thuringiensis* Biofilm

**DOI:** 10.3389/fmicb.2016.01222

**Published:** 2016-08-03

**Authors:** Nay El-Khoury, Racha Majed, Stéphane Perchat, Mireille Kallassy, Didier Lereclus, Michel Gohar

**Affiliations:** ^1^Micalis Institute, Institut National de la Recherche Agronomique (INRA), AgroParisTech, Centre National de la Recherche Scientifique (CNRS), Université Paris-SaclayJouy-en-Josas, France; ^2^Laboratoire de Biotechnologie, Unité de Recherche Technologies et Valorisation Alimentaire, Université Saint-JosephBeirut, Lebanon

**Keywords:** *Bacillus*, thuringiensis, biofilm, phenotypic heterogeneity, sporulation, food microbiology

## Abstract

*Bacillus thuringiensis* can produce a floating biofilm which includes two parts: a ring and a pellicle. The ring is a thick structure which sticks to the culture container, while the pellicle extends over the whole liquid surface and joins the ring. We have followed over time, from 16 to 96 h, sporulation in the two biofilm parts. Sporulation was followed *in situ* in 48-wells polystyrene microtiterplates with a fluorescence binocular stereomicroscope and a spoIID-yfp transcriptional fusion. Sporulation took place much earlier in the ring than in the pellicle. In 20 h-aged biofilms, spoIID was expressed only in the ring, which could be seen as a green fluorescent circle surrounding the non-fluorescent pellicle. However, after 48 h of culture, the pellicle started to express spoIID in specific area corresponding to protrusions, and after 96 h both the ring and the whole pellicle expressed spoIID. Spore counts and microscopy observations of the ring and the pellicle harvested separately confirmed these results and revealed that sporulation occured 24 h-later in the pellicle comparatively to the ring, although both structures contained nearly 100% spores after 96 h of culture. We hypothesize that two mechanisms, due to microenvironments in the biofilm, can explain this difference. First, the ring experiences a decreased concentration of nutrients earlier than the pellicle, because of a lower exchange area with the culture medium. An second, the ring is exposed to partial dryness. Both reasons could speed up sporulation in this biofilm structure. Our results also suggest that spores in the biofilm display a phenotypic heterogeneity. These observations might be of particular significance for the food industry, since the biofilm part sticking to container walls – the ring – is likely to contain spores and will therefore resist both to washing and to cleaning procedures, and will be able to restart a new biofilm when food production has resumed.

## Introduction

Phenotypic heterogeneity in isogenic bacterial populations has been described in several species and for a wide range of phenotypes, including quorum-sensing dependent phenotypes, flagellins expression, response to antibiotics, evasion to host immune response, occurrence of persiter cells, and heat resistance (Abda et al., 2015; Ackermann, 2015; Grote et al., 2015). Stochastic noise in gene expression is thought to be one of the main mechanisms giving rise to this heterogeneity, but a number of other molecular mechanisms can also lead to phenotypic variations (Ackermann, 2015). In *B. subtilis* planktonic cultures, phenotypic variation in sporulation is a consequence of a bistable sporulation gene regulation (Veening et al., 2005).

In biofilms, spatial differentiation is observed in addition to phenotypic heterogeneity. This differentiation is mainly a consequence of the biofilm matrix presence. The biofilm matrix often includes a scaffold of protein fibers (Fong and Yildiz, 2015), and a gel-like structure constituted of polysaccharides (Limoli et al., 2015). The matrix’s low diffusion coefficient limits nutrients exchanges, creates microenvironments within the biofilm, and decreases bacterial motility, which hinders populations remixing. In *B. subtilis* biofilms, the sporulation process was found to occur in specific area described as ‘fruiting bodies’ located at the tip of biofilm protrusions, in which preferential transcription of sporulation genes took place (Branda et al., 2001). Similarly, in colonies grown on agar plates, sporulation, as revealed by transcriptional fusions with reporter genes encoding fluorescent proteins, occurs only in bundles at the upper layers of the colony (Veening et al., 2006). Bacteria located elsewhere in the colony express genes involved in other functions, such as biofilm matrix biosynthesis or motility (Vlamakis et al., 2008).

*Bacillus thuringiensis* is an insect pathogen sharing with *B. subtilis* a number of regulation pathways leading to biofilm formation (Fagerlund et al., 2014). Most strains of this species are able to form biofilms floating on the culture medium (Wijman et al., 2007; Auger et al., 2009). These floating biofilms have a specific architecture, since they include a thick ring which sticks to the container wall and circles the floating pellicle, on which protrusions can be seen (Fagerlund et al., 2014). Floating biofilms of *B. thuringiensis* display a high heterogeneity in genes expression profile, greater than for other growth conditions such as standard agitated cultures, or even during the insect infection (Verplaetse et al., 2015). At least four cell types, which differ within them for the expression of the virulence, necrotrophism, or sporulation regulons, can coexist when the biofilm is produced in an LB-like culture medium (Verplaetse et al., 2015), and three cell types have been observed in HCT, a sporulation culture medium (Verplaetse et al., 2016). Interestingly, sporulation occurs almost exclusively in cells in which the necrotrophism regulon has been activated (Verplaetse et al., 2015). Necrotrophic cells produce a high quantity of degradative enzymes able to degrade the tissues of the host after its death, and therefore to provide the bacterium with nutrients required to achieve the sporulation process (Perchat et al., 2011; Dubois et al., 2012).

Production of spores have been extensively documented in *B. cereus* biofilms (Majed et al., 2016), a species genetically very close to *B. thuringiensis*, from which it differs mainly by the presence in the latter species of Cry plasmids involved in virulence against invertebrates (Jensen et al., 2003). Indeed, spores and biofilms are the main causes of *B. cereus* persistent contamination of industrial food processing lines, which can lead to food spoilage and economical losses, particularly in the dairy industry (Flach et al., 2014). However, the floating biofilm of *B. cereus* or of *B. thuringiensis* have, up to now, always been considered as a whole. Here, we have investigated sporulation in the two main structures of the floating biofilm, the ring and the pellicle. We found differences in the two structures sporulation process, leading to spatial heterogeneity in spore formation in the *B. thuringiensis* biofilm. The consequences of this spatial heterogeneity for the bacterium life and for food industry contamination by *B. cereus* are discussed.

## Materials and Methods

### Strains and Culture Conditions

The strains used in this study were the 407 strain and the 407 pHT304-18ωP*spoIID-yfp* strain. The 407 strain is a *B. thuringiensis* strain cured of its Cry plasmid (Lereclus et al., 1989). The 407 pHT304-18ωP*spoIID-yfp* was obtained after transformation of the 407 strain with the pHT304-18ωP*spoIID-yfp* which construction has been described earlier (Verplaetse et al., 2015). Strains were plated on LB-agar plates and grown overnight at 37°C. One colony from these plates was used to inoculate precultures, of 10 ml of LB medium in 50 ml flasks. Precultures were grown at 30°C with agitation until OD_600_ of 1 was reached. For strain 407 pHT304-18ωP*spoIID-yfp*, the LB agar plate and the preculture medium were supplemented with 10 μg/mL erythromycin.

### *spoIID* Gene Transcription Assay

Transcription assays in biofilms were performed with the 407 pHT304-18ωP*spoIID-yfp* strain (Verplaetse et al., 2015). Precultures were diluted in HCT medium (Lecadet and Martouret, 1967) at a final OD_600_ of 0.01, and 1.5 ml of this solution were distributed in 48-wells polystyrene microtiter plates. The microtiter plates were incubated at 30°C without shaking until observation. Plates were observed after 16, 20, 24, 36, 48, 72, and 96 h of culture with a Leica MZ FLIII fluorescence stereomicroscope (Leica microsystems GmbH, Wetzlar) equipped with a GFP filter (Leica 10446223). Pictures were taken with a CCD camera (Sony NEX5). Fluorescence-white light overlay pictures were obtained with ImageJ 2.0 (Schneider et al., 2012) and annotated or assembled in panels with Photoshop CS6 (Adobe). The wild-type strain displays no autofluorescence background in biofilms produced in HCT (Verplaetse et al., 2016).

### Pellicle and Ring Recovery

**FIGURE 1:**
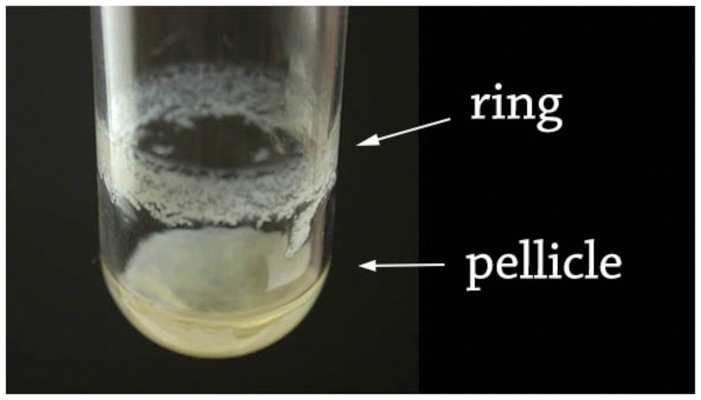
**Ring and pellicle of a 48 h-old *Bacillus thuringiensis* floating biofilm.** The biofilm was formed in a glass tube at 30°C in HCT medium. The culture medium was slowly discarded by pipetting, resulting in a separation of the ring sticking to the tube wall, and of the pellicle laying at the tube bottom.

The 407 wild type strain was grown in glass tubes, in HCT medium at 30°C without shaking, as described elsewhere (Fagerlund et al., 2014). Biofilms were recovered after 16, 20, 24, 48, 72, and 96 h of culture, and the ring and the pellicle were harvested as follows. The culture medium beneath the pellicle was carefully removed with a Pasteur pipette. In this way, the pellicle was detached from the ring, and layed down at the bottom of the tube (**Figure 1**). The pellicle was finally recovered with 1 ml of PBS, and subsequently transferred in a 2 ml microcentrifuge tube. The glass tube without the pellicle was filled with 1ml of PBS, and the ring was scraped with a 1ml pipette cone and recovered in the PBS solution. Both suspensions (the ring and the pellicle) were homogenized by aspirating/pushing ten times through a 26-gauge needle, and their OD_600_ was determined. Suspensions of spores and of vegetative cells display similar absorbances (see **Supplementary Figure S1**), and the OD_600_ is proportional to the number of cells and spores (see **Supplementary Figure S2**).

### Spores Count Assay

Counts of CFU were performed on heated/unheated suspensions by plating serial dilutions triplicates ranging from 10^2^ to 10^7^ spores/ml on LB agar plates, which were subsequently incubated overnight at 30°C. The pellicle and the ring suspensions were heated at 80°C for 12 min to eliminate vegetative cells. Results were expressed as spores percentages relatively to the whole bacterial population.

### Microscope Observations

The presence of spores in the pellicle and the ring fractions of the biofilm were observed with a Zeiss AxioObserver.Z1 microscope, using a phase contrast and oil immersion 100x objective. Pictures were taken with a Zeiss AxioCam MRm CCD camera, annotated with the Zen software (Carl Zeiss Microimaging GmbH, Göttingen) and assembled in panels with Photoshop CS6 (Adobe).

### Statistics

The results are expressed as the mean of 3 to 4 independent experiments, all performed at different days. Means are given with their standard errors.

## Results

### Biofilm Growth

**FIGURE 2:**
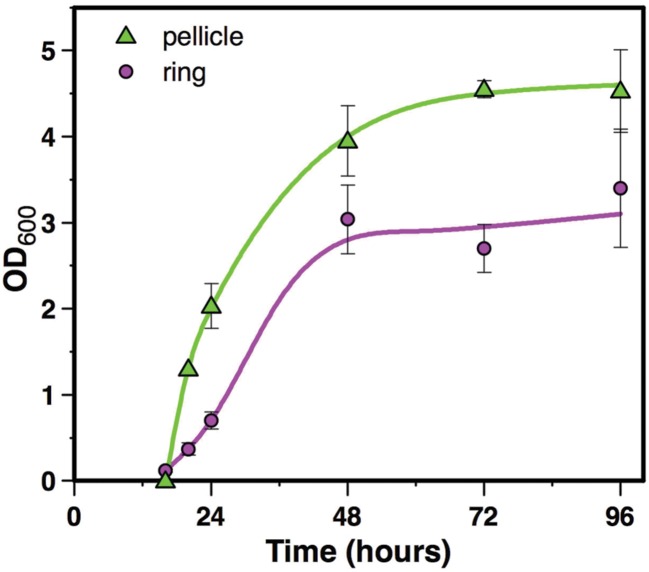
**Biofilm growth.** Biofilm growth in HCT medium at 30°C in glass tubes was recorded by OD_600_ of ring and pellicle suspensions recovered in 1ml water. Symbols represent the mean of 3 to 4 experiments, and bar errors represent the standard error on the mean.

The ring and the pellicle growth curves were determined by the OD_600_ of each fraction suspensions. As depicted **Figure 2**, the ring OD_600_ could be measured as early as after 16 h of culture, while the pellicle OD_600_ could be detected only after 20 h of culture, a time where its structure was stable enough to allow its harvest. After 20 h of culture, the pellicle OD_600_ increased more rapidly than the ring OD_600_, and both growth curves reached a steady state at 48 to 72 h of culture, although the pellicle OD_600_ was 1.7 times higher than the ring OD_600_ during the plateau (between 72 and 96 h). Therefore, the pellicle grew more rapidly than the ring and reached higher OD_600_ values in the mature biofilm.

### Sporulation in the Biofilm

**FIGURE 3:**
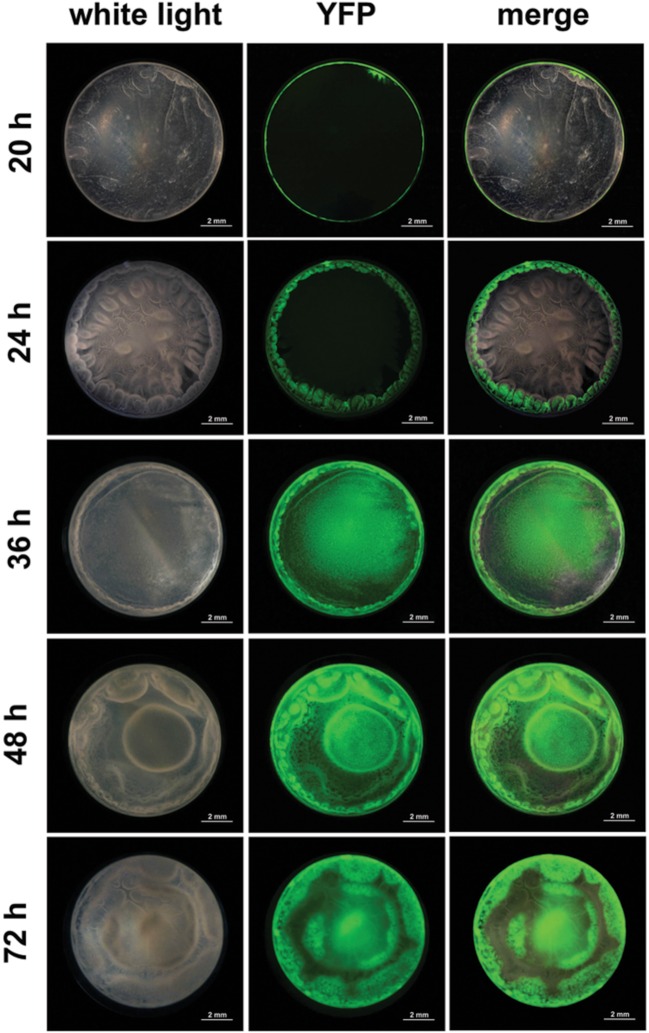
**Sporulation kinetic in a *B. thuringiensis* biofilm.** Biofilms were grown at 30° C in HCT in 48-wells polystyrene microtiter plates. Sporulation was monitored over time (20–72 h) using a *PspoIID-yfp* transcriptional fusion. Top-view pictures of biofilms were taken with a CCD digital camera mounted on a fluorescent stereomicroscope. Pictures were false-colored and merged with the ImageJ software.

Sporulation kinetics, between 16 and 96 h of culture, was determined in the undisturbed floating biofilm formed in 48-wells microtiter plates using a transcriptional fusion between the reporter gene *yfp* and the promoter of *spoIID*, a gene directy controlled by the sigma factor σ^E^ active only when cells are irreversibly engaged into sporulation (Fimlaid and Shen, 2015). After 16 h of culture, the pellicle and the ring were not visible in the biofilm top view, although a faint layer of cells lining the microtiter plate could be seen in a lateral view (not shown). No fluorescence could be observed at this time of the culture, indicating that sporulation in the biofilm had not yet started. Furthermore, the culture medium or the plate did not display any autofluorescence background. Four hours later, at 20 h of culture, a thin pellicle floating on the culture medium surrounded by a ring could be seen (**Figure 3**). A thin circle of fluorescence overlapping the ring was observed and no fluorescence was seen at the pellicle place, showing that sporulation was restricted to the ring at this time of the biofilm growth. After 24 h of culture, the biofilm was well formed and displayed a typical architecture, with a thick ring and a pellicle displaying dense protrusions. Fluorescence overlapped the whole ring but was still absent from the pellicle. However, after 36 h of culture and later on (48 and 72 h), fluorescence had invaded the whole biofilm, although it was, in the pellicle, restricted to specific areas). These results indicated that the sporulation was initiated in the ring long before it started in the pellicle.

### Spore Formation in the Biofilm

**FIGURE 4:**
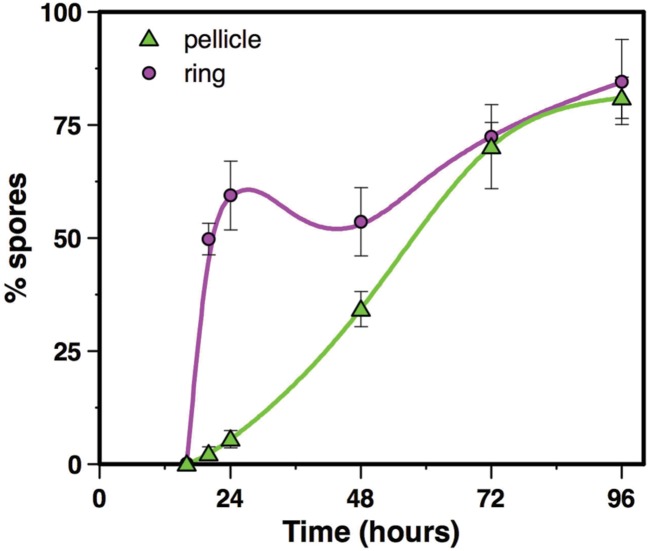
**Spore counts in the ring and in the pellicle.** Biofilms were grown at 30°C in HCT medium in glass tubes, and spore percentages were determined in the ring and the pellicle, between 16 and 96 h of culture. Symbols represent the mean of 3 to 4 experiments, and bar errors represent the standard error on the mean.

**FIGURE 5:**
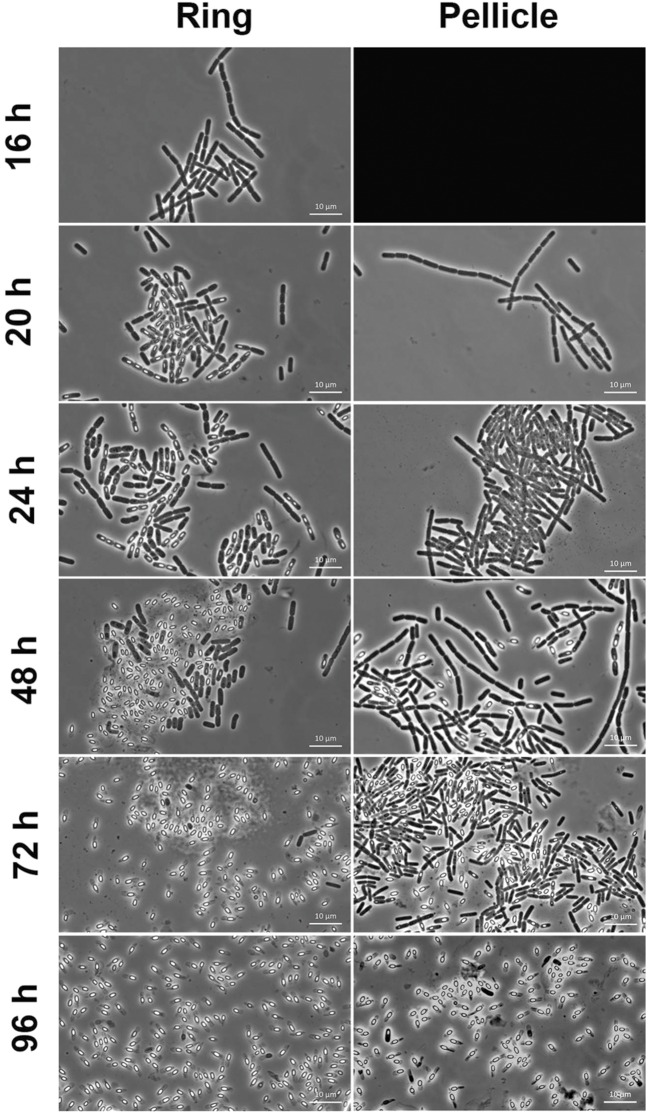
**Microscope observations of the ring and the pellicle.** Biofilms were grown at 30°C in HCT medium in glass tubes, and the ring and the pellicle were harvested at different times between 16 and 96 h of culture, and observed in a microscope for spores and vegetative cells. The pellicle could not be harvested at 16 h, and its image is replaced by a black background.

Spores count in the pellicle was very low (below 5.5% spores) before 24 h of culture and increased slowly thereafter to reach 34% spores after 48 h of culture and 81% after 96 h of culture (**Figure 4**). In contrast, spores count in the ring, which level was at 0.2% after 16 h of culture, increased steeply to reach 60% after 24 h of culture (**Figure 4**). The percentage of spores in the ring decreased slightly after that time, between 24 and 48 h of culture, and increased again after 48 h of culture, to parallel the pellicle spores count curve between 72 and 96 h of culture (**Figure 4**). Thus, there is at least a 24 h-delay between the ring and the pellicle for spore formation. We then used microscope observations of the resuspended biofilm to confirm this result. In the ring harvested at 16 h of culture only vegetative cells without spores were present (**Figure 5**). However, after 20 or 24 h of culture, about 50% of the ring vegetative cells included spores, whereas almost no spores could be observed inside the pellicle vegetative cells. Spores were not seen in the pellicle until 48 h of culture, a time at which they represented less than half of the bacteria, whereas a high number of free spores could be observed in the ring at that time (**Figure 5**). After 72 h of culture and later, spores made nearly 100% of bacteria in the ring, while in the pellicle the percentage of spores increased from about 60% after 72 h to 80% after 96 h (**Figure 5**).

## Discussion

Despite similar growth curve shapes displayed by the *B. thuringiensis* biofilm ring and pellicle, this study showed that the two structures behaved quite differently regarding the sporulation process. In the ring, sporulation could be observed after 20 h of culture, and more than half of the ring bacterial population had already sporulated after 24 h of culture. In contrast, sporulation in the pellicle biofilm started after 36 h and resulted in about 35% of spores after 48 h. Thus, the sporulation kinetic was delayed by more than 24 h in the pellicle comparatively to the ring. Early nutrients deprivation in the ring compared to the pellicle could explain this difference in the sporulation kinetics of the two structures. Indeed, while the pellicle is thin and bathed by the culture medium on half of its surface, the ring is thick and in contact with the culture medium on roughly 1/4th of its surface, thus limiting nutrients exchanges. In addition, the ring is submitted to partial dryness because it extends above the culture medium surface as a consequence of two phenomenons. First, a meniscus at the air-glass-liquid interface allows an initial adhesion of bacterial cells above the average culture medium surface. Second, a slight evaporation decreases the liquid height in the glass tube as time elapses – but the ring, which sticks to the tube walls, stays in the same place. Dryness has been shown to favor sporulation in *B. cereus* biofilms (Ryu and Beuchat, 2005; Hayrapetyan et al., 2016) and could therefore play a role in the early sporulation of the ring.

**FIGURE 6:**
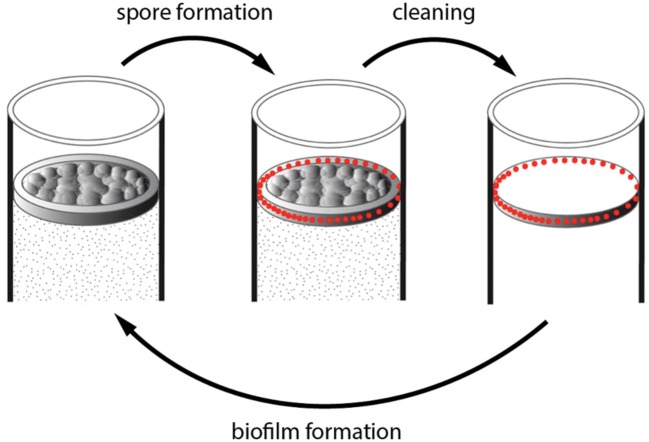
**Model of *B. thuringiensis* biofilm resistance against cleaning.** A biofilm is formed in a tank containing a medium suitable for its growth (left drawing). Spores (red dots) are formed preferentially in the ring (up left arrow). The mechanical washing procedure can discard the pellicle but the ring, which sticks to the tank wall, stays in place (up right arrow). Spores in the ring located on the container wall can resist the chemical cleaning procedure, and will germinate when conditions become suitable, and give birth to a new biofilm (bottom arrow).

The early sporulation in the ring – representing about 2/3rd of the pellicle for the number of cells – might warrant the bacterial population survival in case of changes in the environmental conditions, while the delayed sporulation in the pellicle would allow a continuation in the bacterial population growth – reminding bet-hedging strategies found in several cases of phenotypic heterogeneity (van Gestel et al., 2015). In food industry equipments, for instance, environmental changes are frequent. Biofilm formed in these equipments are regularly submitted to high pressure washing followed by chemical cleaning procedures (Majed et al., 2016). The biofilm ring, which adheres strongly to the recipient walls, is likely to remain in place after the washing procedures while the pellicle will be discarded. Spores in the ring will be resistant to the cleaning procedure, and will germinate and give birth to a new biofilm when conditions are suitable again (**Figure 6**).

While the ring and the pellicle spore percentage of 96 h -aged biofilms was around 80% when measured by plating, this percentage was close to 100% according to microscopy observations. This difference can be explained if a proportion of the spores found in the biofilm exhibit a decreased resistance to heat, suggesting that phenotypic heterogeneity is also displayed in the properties of biofilm spores. Spores from planktonic cultures were shown to be less heat resistant compared to spores from biofilms (Veening et al., 2006), and other spore properties, such as resistance to various stresses or sensitivity to germinants, are dependent on the culture conditions met by bacteria during sporulation (Hornstra et al., 2006; Broussolle et al., 2008; Planchon et al., 2011). As a consequence, spores produced in the biofilm ring could be different for their properties from spores produced in the biofilm pellicle.

The slight decrease in the ring spore counts at 48 h of culture, compared to 24 h of culture, has been observed earlier in a microtiter plate assay for the *B. cereus* strain ATCC10987 (Hayrapetyan et al., 2016). This decrease is not subsequent to a decrease in the biofilm biomass, since both the biofilm ring and pellicle biomasses increase continuously with time. It might rather be due to a transient and limited germination of spores in the ring. Microscope images show that vegetative cells are still present in the ring at 48 h of culture, although in small proportion. This germination could be consecutive to cell lysis in a fraction of the ring population, which would deliver germinants to spores.

## Author Contributions

NE-K performed the work and wrote the manuscript RM and SP performed the work and designed experiments MK and DL designed experiments MG designed experiments and wrote the manuscript.

## Conflict of Interest Statement

The authors declare that the research was conducted in the absence of any commercial or financial relationships that could be construed as a potential conflict of interest.
