# Supplementary figures and images for: Spatio-Temporal Evolution of Sporulation in Bacillus thuringiensis Biofilm
Source: Front Microbiol. 2016 Aug 3;7:1222. doi: 10.3389/fmicb.2016.01222 (PMC4971082; doi:10.3389/fmicb.2016.01222)

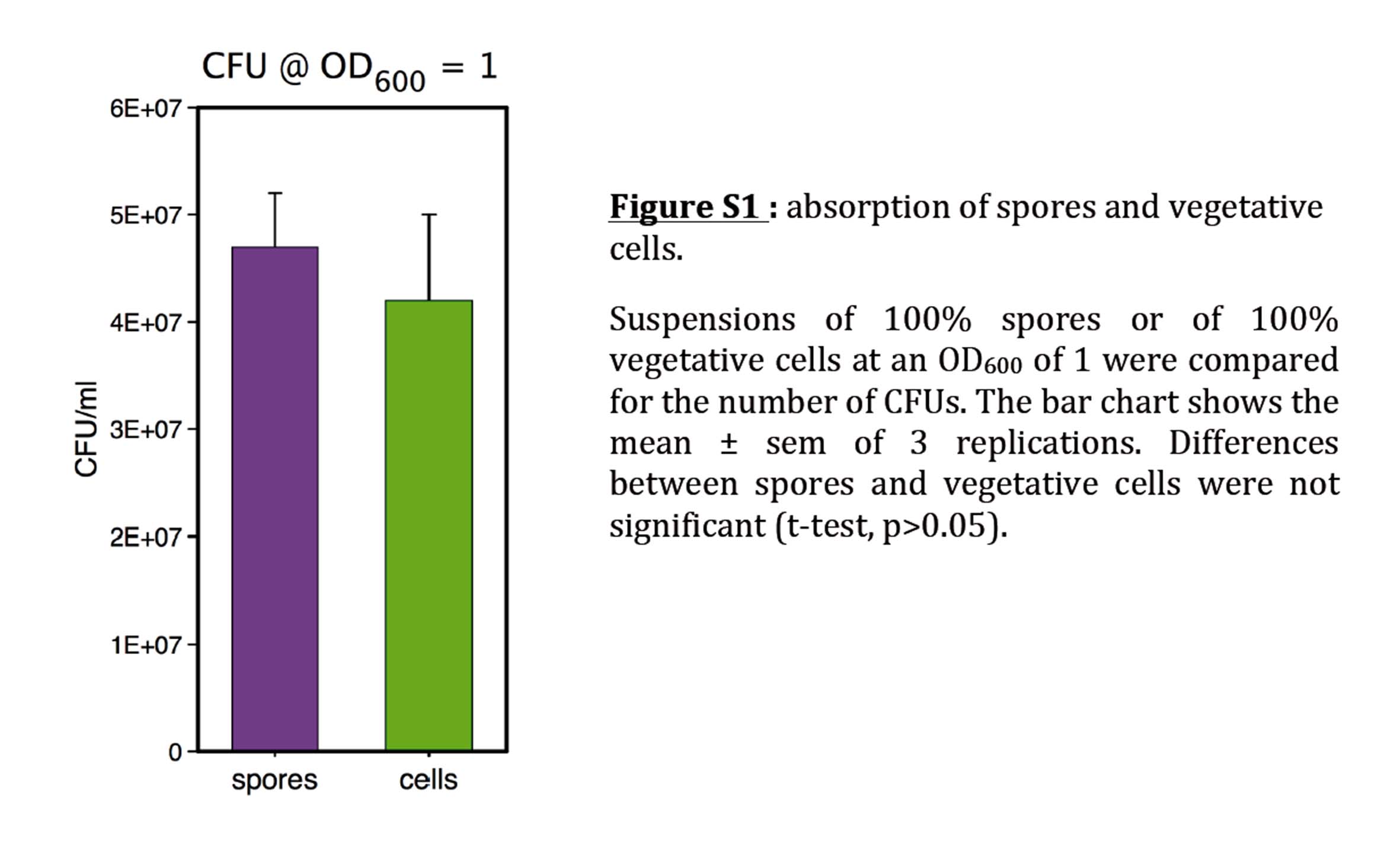

Supplement: Supplementary file 1 [file Image_1.JPEG]

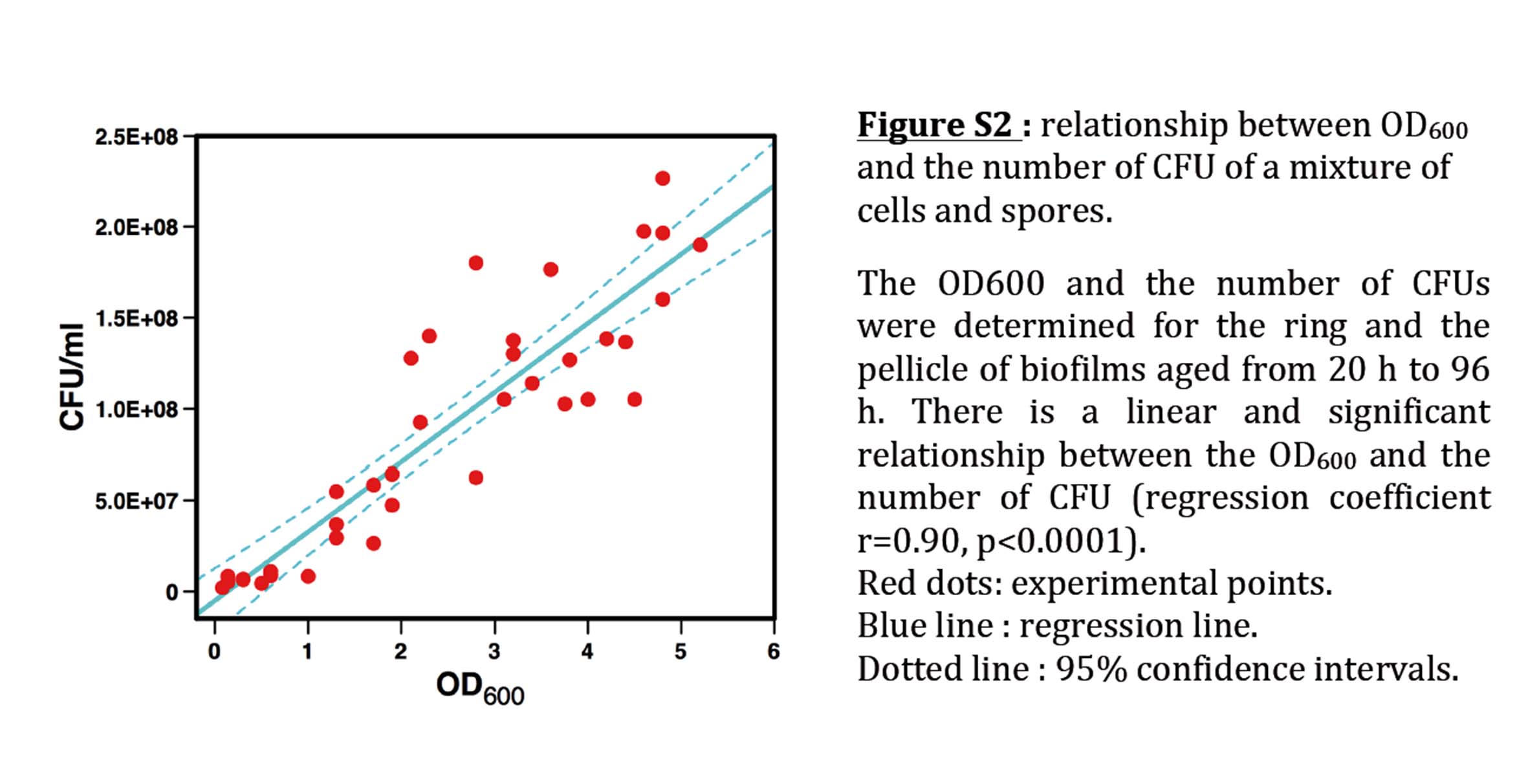

Supplement: Supplementary file 2 [file Image_2.JPEG]
